# Unveiling the truth: Pathogen infections linked to miscarriage: A STROBE-Compliant Mendelian randomization study

**DOI:** 10.1097/MD.0000000000040627

**Published:** 2024-11-22

**Authors:** Jie Zhou, Jiekai Yin, Yixin Xu, Haitao Wang

**Affiliations:** a Department of General Surgery, The Wujin Hospital Affiliated with Jiangsu University, Changzhou, China; b Department of General Surgery, The Wujin Clinical College of Xuzhou Medical University, Changzhou, China; c Department of Emergency Medicine, The Wujin Clinical College of Xuzhou Medical University, Changzhou, China; d Department of General Surgery, The Third Affiliated Hospital of Soochow University, Changzhou, China.

**Keywords:** antibody-mediated immune response, causal relationship, genetically predicted, GWAS, single nucleotide polymorphism

## Abstract

Miscarriage represents a prevalent yet insufficiently studied adverse pregnancy outcome. The definitive causal links between various pathogens and miscarriage remain to be established. To investigate the causal connections between pathogen infections and miscarriage, we utilized a two-sample bidirectional Mendelian randomization (MR) analysis. We sourced genome-wide association studies data on pathogen infections from the UK Biobank, which included serological markers for infectious diseases and comprehensive whole-genome genetic information from approximately 10,000 individuals. Additionally, genome-wide association studies data on miscarriages were collected from 3 distinct European populations for our analysis. The MR analysis was primarily conducted using the inverse variance weighted method, complemented by Bayesian weighted MR and the weighted median method for robustness. To ensure the reliability of our findings, we performed heterogeneity and pleiotropy tests, leave-one-out sensitivity analyses, and a meta-analysis. Our extensive research has identified a causal association between miscarriage and infections by several human herpesviruses (HHV-1, HHV-3, HHV-4, HHV-6, and HHV-7), polyomaviruses (BK, JC, and Merkel cell polyomaviruses), and *Chlamydia trachomatis* (inverse variance weighted, *P* < .05). Notably, a meta-analysis of the integrated data highlighted the particularly high accuracy and consistency of the association with Merkel cell polyomavirus. Our MR analysis has clarified the causal relationships between specific pathogen infections and miscarriage, providing a critical foundation for the prevention and treatment of this adverse pregnancy outcome.

## 1. Introduction

Miscarriage is generally defined as the loss of a pregnancy before viability.^[1]^ Based on current data, among 1.3 billion newborns worldwide, the overall risk of miscarriage in confirmed pregnancies is 15.3% (95% confidence interval: 12.5–18.7%). A 15% risk of miscarriage implies approximately 23 million cases per year, or 44 miscarriages every minute.^[2]^ While miscarriage typically does not have a significant impact on women’s health, some patients may experience pain, bleeding, and a risk of hemorrhage. Importantly, feelings of loss and sadness are common, and the psychological well-being of those affected may be affected.^[3]^ As a common pregnancy complication that impacts every family, there is an urgent need for more research to deepen our understanding of its effects and preventive measures.

The etiology of miscarriage remains incompletely understood. Over 60% of early pregnancy losses are attributed to fetal chromosomal abnormalities,^[4]^ with advanced parental age being a contributing factor.^[5]^ Additionally, anatomical factors, immune dysregulation, endocrine factors, nutritional/metabolic factors, and unhealthy lifestyle habits are also considered risk factors for miscarriage.^[4,6]^ It is worth noting that many infections have been found to be associated with miscarriage.^[7]^ Notably, 15% of early miscarriages and 66% of late miscarriages are attributed to infections.^[8,9]^

The causal relationship between different pathogens and miscarriage has not yet been conclusively established.^[10]^ For example, there are conflicting research findings regarding the impact of *Chlamydia trachomatis* infection on miscarriage. Some studies suggest that *C trachomatis* infection may be associated with miscarriage,^[11–14]^ as seen in the study by Baud et al, which found higher levels of *C trachomatis* antibodies in the miscarriage group.^[11]^ However, research by Feist et al indicates that there is no clear association between *C trachomatis* infection and miscarriage.^[15]^ The role of herpes simplex virus (HSV) in miscarriage remains uncertain.^[10]^ While studies have detected the DNA of both HSV-1 and HSV-2 in miscarriage tissues, the lack of differentiation between the 2 strains may introduce ambiguity regarding their specific impact on miscarriage.^[16]^ Additionally, in a cross-sectional study conducted by Kim et al, the prevalence of HSV-2 seropositivity was higher in the miscarriage group. However, due to the presence of numerous confounding factors, establishing a causal relationship was not feasible.^[17]^ Observational studies suggest a potential association between *Toxoplasma gondii* infection and miscarriage, yet the absence of control group data necessitates further research to validate this relationship.^[18–20]^ Similarly, the detection of BK polyomavirus (BKPyV) in specimens from miscarriages associated with chorioamnionitis is limited by the extremely small sample size (n = 5), thus restricting the reliability of the conclusions.^[21,22]^

To overcome the limitations of these studies, such as unmeasured or imprecisely measured confounders, reverse causation, and other sources of bias, the use of genome-wide association studies (GWAS) data for Mendelian randomization (MR) has emerged as a promising method for evaluating causal relationships in assumed exposure-outcome pathways.^[23]^ Essentially, MR acts as a natural randomized trial, utilizing the random allocation of genetic variants at conception to partition individuals into different subgroups, akin to a placebo group and an intervention group in a randomized controlled trial. This method enables the evaluation of the potential causal relationship between risk factors (pathogen infections) and disease outcomes (miscarriage), while ensuring the randomness of confounding variables.^[24]^ In our research, we compiled recent GWAS summaries on pathogen infections and miscarriage. Through a two-sample MR analysis, our primary objective is to understand the causal relationship between pathogen infections and miscarriage, with the ultimate goal of developing more effective prevention and treatment strategies, recognizing the priceless value of life.

## 2. Methods

### 2.1. Study design

In this study, all data were obtained from publicly available databases and received approval from the relevant research institution’s review board, thereby bypassing the need for ethical committee review.

Our research employed single nucleotide polymorphisms (SNPs) as instrumental variables (IVs) to conduct a comprehensive bidirectional MR analysis, investigating the causal relationship between pathogen infections and miscarriage.^[25]^ Additionally, the IVs included in the MR analysis must adhere to 3 critical assumptions: (1) SNPs are associated with the exposure; (2) SNPs are independent of confounding factors in the exposure–outcome relationship; (3) SNPs solely influence the outcome through the exposure.^[26]^

### 2.2. GWAS summary data sources

#### 2.2.1. Data for pathogen infection

In our study, data on pathogen infections were reflected through the measurement of infections and antibody-mediated immune responses (IAMIR). The GWAS data on IAMIR used in this study were obtained from the UK Biobank, including measurements of infectious disease serology and whole-genome genetic typing from up to 10,000 individuals.^[27]^ The research team utilized data from 13 pathogens and defined 46 phenotypes, including 15 seropositive case–control phenotypes and 31 antibody measurement phenotypes (Table S1, Supplemental Digital Content, http://links.lww.com/MD/O6). The study encompassed 13 infectious agents, including HSV-1, HSV-2, Epstein-Barr virus (EB virus), human cytomegalovirus, human herpesvirus-6, human herpesvirus-7, varicella zoster virus, human BKPyV, human JC polyomavirus (JCPyV), Merkel cell polyomavirus (MCPyV), *C trachomatis*, *Helicobacter pylori*, and *T gondii*.

It is worth noting that many of the aforementioned infectious agents have been subject to significant controversy regarding their causal relationship with miscarriage, such as *C trachomatis*, *T gondii*, HSV, and human BKPyV. This controversy makes them particularly suitable for inclusion in this study.

#### 2.2.2. Data for miscarriage

To bolster the credibility of our study, we have incorporated 3 expansive GWAS datasets for a comprehensive joint analysis. Firstly, 1 dataset sourced from the FinnGen database encompasses 18,680 cases of miscarriage and 162,987 control subjects (Phenocode: O15_ABORT_SPONTAN). Furthermore, we have leveraged 2 additional GWAS datasets concerning miscarriage from the IEU OpenGWAS project (mrcieu.ac.uk). The GWAS dataset identified by the ID ebi-a-GCST011888 (analyzed by Triin Laisk et al) is composed of European populations, comprising 49,996 cases and 174,109 controls.^[28]^ Similarly, the GWAS dataset with the ID ebi-a-GCST90018786 (analyzed by Saori Sakaue et al) features European populations, encompassing 7069 miscarriage cases and 250,492 control individuals.^[29]^ The detailed information regarding the aforementioned data is outlined in Table 1.

**Table 1:** Details of the genome-wide association studies and datasets used in our analyses.

Phenotypes	Cases/controls	Consortium/author	Population	PubMed ID	Data download link
Spontaneous abortion	18,680/162,987	FinnGen consortium	European	–	https://storage.googleapis.com/finngen-public-data-r10/summary_stats/finngen_R10_O15_ABORT_SPONTAN.gz
Sporadic miscarriage	49,996/174,109	Triin Laisk et al	European	33239672	https://www.ebi.ac.uk/gwas/; ID, ebi-a-GCST011888
Abortion	7069/250,492	Saori Sakaue et al	European	34594039	https://www.ebi.ac.uk/gwas/; ID, ebi-a-GCST90018786
IAMIR	8735	UK Biobank	European	33204752	https://www.ebi.ac.uk/gwas/; Accession numbers GCST90006884–GCST90006929

### 2.3. IVs selection and data harmonization

In our study, we meticulously screened SNPs using stringent criteria to ensure the robustness of our conclusions. To guarantee a sufficient number of IVs, we selected SNPs with genome-wide significance (*P* < 5 × 10^‐6^) for further investigation. Moreover, we excluded palindromic and ambiguous SNPs as IVs in our analysis.^[30]^ Subsequently, we grouped SNPs based on linkage disequilibrium using a window size of 10,000 kb and an *r*^2^ threshold of <0.001. Additionally, we calculated the *F* statistic using the formula [(N ‐ K ‐ 1)/K]/[*R*^2^/ (1 ‐ *R*^2^)] to evaluate the variance explained by each exposed SNP, where *K* denotes the number of genetic tools and *N* represents the sample size.^[31]^ To ensure the reliability and consistency of our findings, we eliminated weak IVs with *F* < 10. Furthermore, we conducted a comprehensive literature review to assess all established phenotypes related to the genetic tools considered in our study, meticulously excluding SNPs associated with confounding factors.

### 2.4. Statistical analysis

In our thorough and comprehensive analysis, we utilized R software (version 4.2.0, http://www.r-project.org) in combination with the “Two-Sample MR” package (version 0.5.6) to perform MR analysis.^[32]^

### 2.5. Primary analysis

To investigate the correlation between IAMIR and miscarriage, we conducted a two-sample MR analysis. In this study, we utilized the GWAS data for IAMIR as the exposure and the GWAS data for 3 groups of miscarriage as the outcome. Our primary analytical method, the inverse variance weighted (IVW) method, combines a meta-analysis strategy with the Wald estimate of each SNP. The IVW results remain unbiased in the absence of horizontal pleiotropy.^[33]^ A significance level of *P* < .05 indicates a positive result. In addition to the IVW method, we also incorporated supplementary approaches such as Bayesian weighted MR^[34]^ and the weighted median method.^[35]^ Specifically, the Bayesian weighted MR method is tailored for causal inference, tackling uncertainties stemming from weak effects due to polygenicity. It achieves this by employing Bayesian weighting to identify outliers and manage violations of the IV assumption arising from pleiotropy. The weighted median method showcases enhanced precision, evident in its smaller standard deviation compared to MR-Egger analysis. Even in the presence of horizontal pleiotropy, the weighted median method delivers a consistent estimate, even when considering 50% of genetic variants as invalid instrumental variables.^[36]^

Following a comprehensive analysis, we will extensively showcase all positive outcomes derived from the 3 sets of MR analyses in the results section. Initially, based on the results obtained using the IVW method, we will proceed to consolidate all positive phenotypes and compare them with 3 sets of miscarriage GWAS data. Subsequently, we will conduct a meta-analysis using the “meta” package to delve deeper into the degree of association between various IAMIR phenotypes and miscarriage. We will utilize the *I*^2^ statistic to quantify the level of inconsistency within studies, which delineates the proportion of heterogeneity. In the absence of heterogeneity, we will opt for a common effect model to calculate the overall odds ratio (OR); otherwise, a random-effects model will be employed.

### 2.6. Reverse MR analysis

To explore the potential reverse causal relationship between IAMIR and miscarriage, this study conducted a reverse MR analysis, with miscarriage as the exposure and previously identified positive IAMIR phenotypes as the outcome. The aforementioned methods were also utilized for the analysis, and the meta-analysis will further validate the reliability of the conclusions drawn from the reverse MR analysis.

### 2.7. Sensitivity analysis

Due to variations in experimental conditions, study populations, and SNPs, heterogeneity can arise during two-sample MR analysis, potentially introducing biases in estimating causal effects. Therefore, this study conducted heterogeneity assessments using both the IVW and MR-Egger methods. Cochrane Q statistic was employed to evaluate the heterogeneity of genetic instruments, with a *P*-value > .05 indicating non-significant heterogeneity.^[37]^ A fundamental assumption in MR analysis is that the IV influences the outcome solely through the exposure, necessitating an examination of potential horizontal pleiotropy between the exposure and outcome.^[38]^ The study utilized the MR-Egger intercept method to assess the presence of pleiotropy, where a *P*-value above .05 suggests minimal or negligible potential for pleiotropy in causal analysis, allowing for its exclusion. Outliers identified in the IVW analysis were identified and adjusted using the MR-PRESSO test.^[39]^ Finally, the leave-one-out analysis was used to determine the genetic causal effects of individual SNPs on the exposure–outcome relationship.^[40]^

## 3. Results

**Figure 1.**
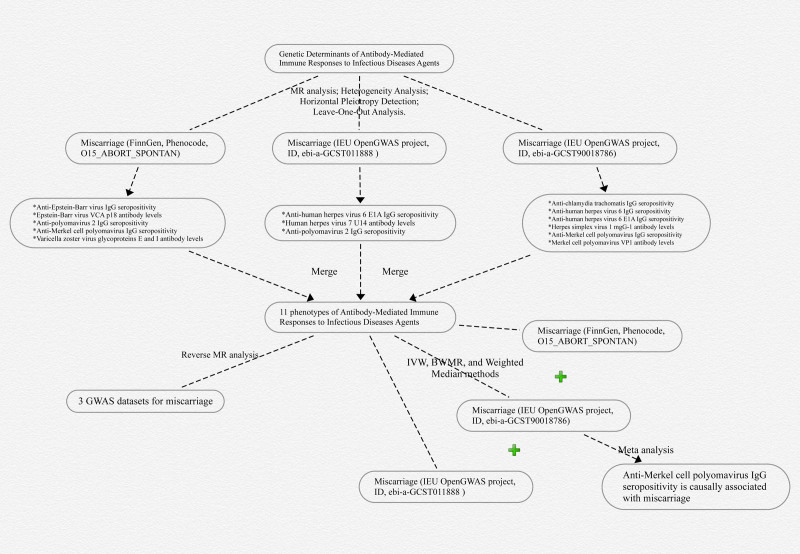
The schematic representation of the study design. GWAS = genome-wide association studies; MR = Mendelian randomization.

The schematic representation of the study design can be found in Figure 1.

### 3.1. Association of 46 phenotypes of IAMIR and miscarriage

To investigate the genetically predicted causal relationship between the IAMIR and miscarriage, we treated 46 phenotypes of IAMIR as exposures and 3 groups of miscarriage as the outcome.

**Figure 2.**
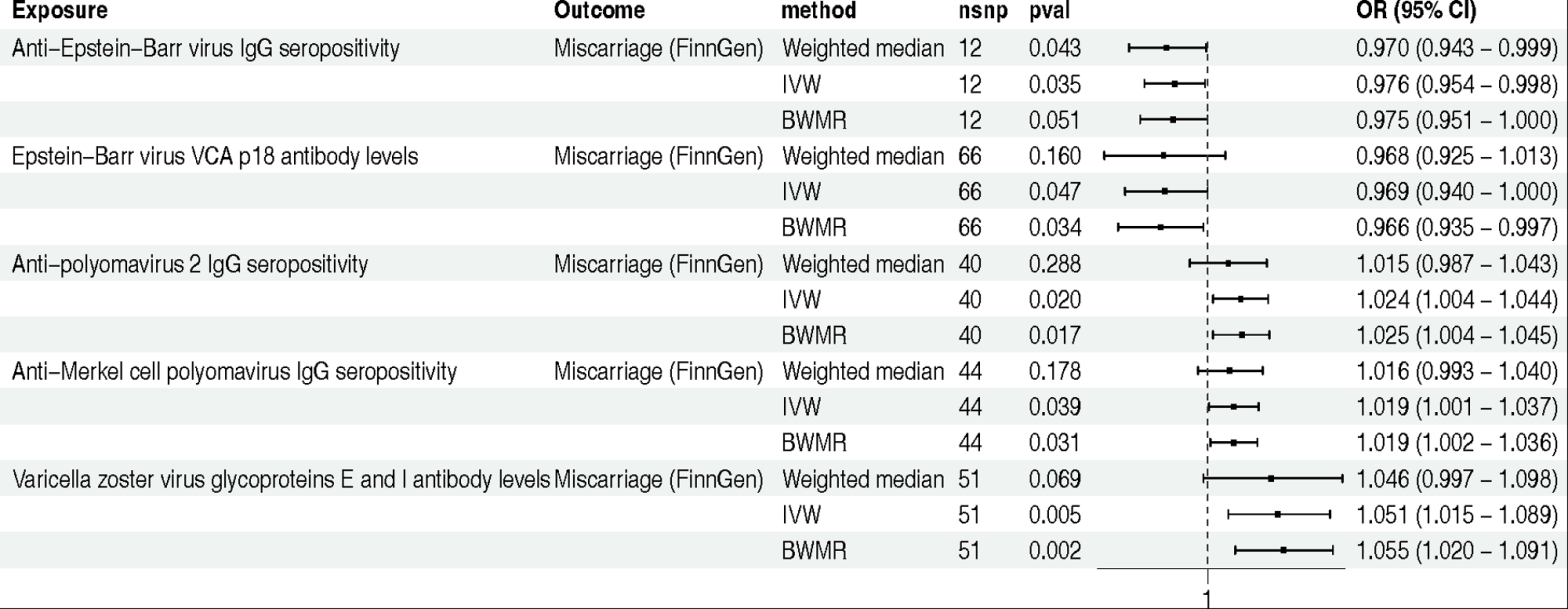
The MR analysis between IAMIR and miscarriage (FinnGen). *Note*: The GWAS data for miscarriage was derived the FinnGen consortium. BWMR = Bayesian weighted Mendelian randomization; CI = confidence interval; IAMIR = infections and antibody-mediated immune responses; IVW = inverse variance weighted; MR = Mendelian randomization; nSNPs = number of single nucleotide polymorphisms; OR = odds ratio.

In the initial analysis using GWAS data on miscarriage from the Finnish database, a total of 6 phenotypes were identified to exhibit a causal relationship with miscarriage following screening with the IVW method. Subsequent analyses on heterogeneity and horizontal pleiotropy led to the exclusion of Epstein-Barr virus ZEBRA antibody levels. The remaining 5 phenotypes include: anti-Epstein-Barr virus IgG seropositivity (IVW method: OR 0.976; 95% confidence interval [CI] 0.954–0.998; *P* = .035), Epstein-Barr virus VCA p18 antibody levels (IVW method: OR 0.969; 95% CI 0.940–1.000; *P* = .047), anti-polyomavirus 2 IgG seropositivity (IVW method: OR 1.024; 95% CI 1.004–1.044; *P* = .020), anti-Merkel cell polyomavirus IgG seropositivity (IVW method: OR 1.019; 95% CI 1.001–1.037; *P* = .039), and varicella zoster virus glycoproteins E and I antibody levels (IVW method: OR 1.051; 95% CI 1.015–1.089; *P* = .005) (Fig. 2).

**Figure 3.**
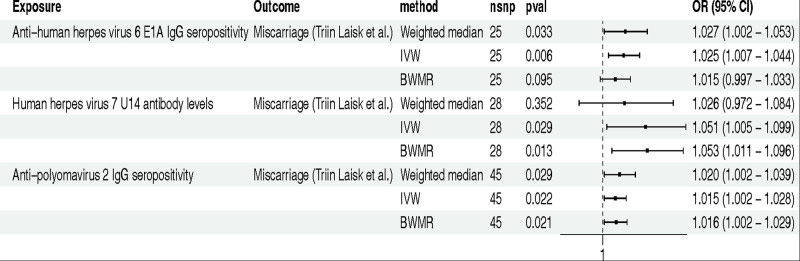
The MR analysis between IAMIR and miscarriage (Triin Laisk et al). *Note*: The GWAS data for miscarriage was analyzed by Triin Laisk et al. BWMR = Bayesian weighted Mendelian randomization; CI = confidence interval; IAMIR = infections and antibody-mediated immune responses; IVW = inverse variance weighted; MR = Mendelian randomization; nSNPs = number of single nucleotide polymorphisms; OR = odds ratio.

In the GWAS data on miscarriage analyzed by Triin Laisk et al, we conducted a meticulous analysis employing similar methodologies. The findings revealed causal relationships between miscarriage and anti-human herpes virus 6 E1A IgG seropositivity (IVW method: OR 1.025; 95% CI 1.007–1.044; *P* = .006), human herpes virus 7 U14 antibody levels (IVW method: OR 1.051; 95% CI 1.005–1.099; *P* = .029), as well as anti-polyomavirus 2 IgG seropositivity (IVW method: OR 1.015; 95% CI 1.002–1.028; *P* = .022) (Fig. 3).

**Figure 4.**
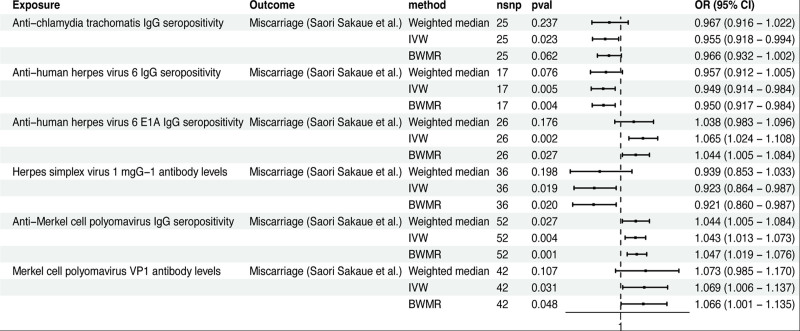
The MR analysis between IAMIR and miscarriage (Saori Sakaue et al). *Note*: The GWAS data for miscarriage was analyzed by Saori Sakaue et al. BWMR = Bayesian weighted Mendelian randomization; CI = confidence interval; IAMIR = infections and antibody-mediated immune responses; IVW = inverse variance weighted; MR = Mendelian randomization; nSNPs = number of single nucleotide polymorphisms; OR = odds ratio.

Similarly, in the GWAS data on miscarriage analyzed by Saori Sakaue et al, 6 phenotypes were found to be causally associated with miscarriage. These are anti-*Chlamydia trachomatis* IgG seropositivity (IVW method: OR 1.025; 95% CI 1.007–1.044; *P* = .006), anti-human herpes virus 6 IgG seropositivity (IVW method: OR 0.949; 95% CI 0.914–0.984; *P* = .005), anti-human herpes virus 6 E1A IgG seropositivity (IVW method: OR 1.065; 95% CI 1.024–1.108; *P* = .002), herpes simplex virus 1 IgG antibody levels (IVW method: OR 0.923; 95% CI 0.864–0.987; *P* = .019), anti-Merkel cell polyomavirus IgG seropositivity (IVW method: OR 1.043; 95% CI 1.013–1.073; *P* = .004), and Merkel cell polyomavirus VP1 antibody levels (IVW method: OR 1.069; 95% CI 1.006–1.137; *P* = .031) (Fig. 4).

It is worth noting that all the relevant information of the SNPs (Table S2, Supplemental Digital Content, http://links.lww.com/MD/O6) and the complete results of the MR analysis (Table S3, Supplemental Digital Content, http://links.lww.com/MD/O6) mentioned above can be found in the supplementary files. Moreover, all MR analyses conducted were devoid of heterogeneity and horizontal pleiotropy (Table S6, Supplemental Digital Content, http://links.lww.com/MD/O6). During outlier detection, no anomalies were detected. Lastly, employing a “leave-one-out” sensitivity analysis approach revealed that the systematic exclusion of each SNP did not substantially alter the model’s effect estimates or qualitative conclusions (Figs. S1, Supplemental Digital Content, http://links.lww.com/MD/O5, S2, Supplemental Digital Content, http://links.lww.com/MD/O5, and S3, Supplemental Digital Content, http://links.lww.com/MD/O5).

### 3.2. Data integration and meta-analysis

Based on the summary of the above results, we identified 11 phenotypes that may be causally associated with miscarriage. In these phenotypes, anti-human herpes virus 6 E1A IgG seropositivity, anti-polyomavirus 2 IgG seropositivity, and anti-Merkel cell polyomavirus IgG seropositivity showed significance in 2 out of the 3 MR analyses conducted. To improve the reliability of the results, we recorded the results of IVW analysis for each of these 11 phenotypes in the 3 sets of MR analyses (Table S4, Supplemental Digital Content, http://links.lww.com/MD/O6) and performed meta-analysis.

All the meta-analysis results are provided in the supplementary files. It is noteworthy that the anti-polyomavirus 2 IgG seropositivity, as an exposure factor, demonstrates a causal relationship with miscarriage as analyzed by Triin Laisk et al and in the FinnGen dataset. However, in the MR analysis conducted with the miscarriage GWAS data analyzed by Saori Sakaue et al, the results exhibit horizontal pleiotropy, thereby precluding meta-analysis. The potential causal relationship between anti-polyomavirus 2 IgG seropositivity and miscarriage is significant and deserves attention from clinical practitioners. However, further validation with additional data is necessary in subsequent studies.

**Figure 5.**
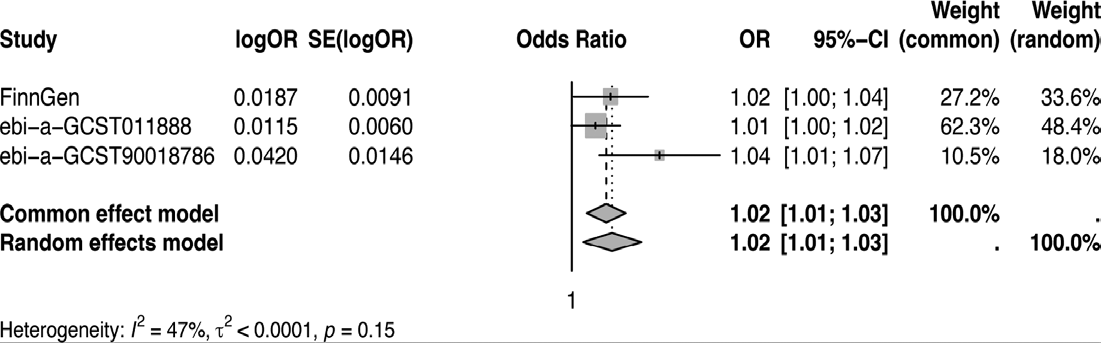
A meta-analysis of anti-Merkel cell polyomavirus IgG seropositivity as a risk factor for miscarriage. CI = confidence interval; OR = odds ratio.

Furthermore, it was found in the meta-analysis that there is a causal relationship between anti-Merkel cell polyomavirus IgG seropositivity and miscarriage (OR 1.02; 95% CI 1.01–1.03; *P* = .006) (Fig. 5).

### 3.3. Reverse MR analysis

To explore whether there is a reverse causal relationship between the aforementioned 11 phenotypes and miscarriage, we conducted reverse MR analysis using these 11 phenotypes as outcomes and GWAS data for 3 groups of miscarriage as exposures (Table S5, Supplemental Digital Content, http://links.lww.com/MD/O6). When utilizing the miscarriage GWAS data from the FinnGen dataset, we did not find any significant associations with any of the aforementioned phenotypes. Similarly, when using the miscarriage data analyzed by Triin Laisk et al, no causal relationships between the 2 were identified.

However, in the GWAS data analysis conducted by Saori Sakaue et al, a causal relationship was found between miscarriage and anti-polyomavirus 2 IgG seropositivity (IVW method: OR 1.239; 95% CI 1.000–1.535; *P* = .0498), with no evidence of horizontal pleiotropy or heterogeneity (Table S6, Supplemental Digital Content, http://links.lww.com/MD/O6). However, this result is somewhat close to the significance threshold and has not been validated in other datasets. Therefore, further research is needed to confirm the stability and reliability of the findings.

## 4. Discussion

Miscarriage is one of the most common yet understudied adverse pregnancy outcomes, causing significant pain and psychological burden to many families. According to statistics, there are 23.99 million cases of miscarriage annually, with an overall miscarriage risk during pregnancy of approximately 15.3%.^[2]^ While numerous cross-sectional studies have begun to explore the relationship between bacterial, viral, and parasitic infection factors and the risk of miscarriage, the current conclusions are still subject to significant controversy due to issues such as inadequate sample sizes and numerous confounding factors.^[10]^ To the best of our knowledge, this is the first study to systematically explore the potential causal relationships between different pathogen infections and miscarriage using two-sample MR. This MR analysis is based on robust IVs from a large-scale GWAS database of European populations. To enhance the accuracy of the results, this study incorporated GWAS data from 3 sets of miscarriages for analysis and synthesis. The study ultimately revealed causal relationships between certain pathogen infections and miscarriage, contributing to a deeper understanding of the complex relationship between pathogen infections and miscarriage.

### 4.1. Viral infections

#### 4.1.1. Herpes virus infections

The herpes family of DNA viruses comprises numerous human pathogenic viruses capable of remaining latent in the host and later reactivating.^[41]^ This study conducted a comprehensive analysis on the correlation between 7 herpesviruses and miscarriage, uncovering a plausible causal relationship between different herpesvirus infections and miscarriage. Through the integrated analysis of GWAS data from various miscarriage datasets, notable levels of anti-human herpes virus 6 E1A IgG seropositivity were identified in 2 datasets. However, this significance did not persist in the subsequent meta-analysis.

The research conducted by Kapranos and Kotronias unveiled higher positivity rates of HSV-1 and HSV-2 in spontaneous miscarriage samples than in selective miscarriage samples. Nevertheless, due to the lack of differentiation between HSV-1 and HSV-2, it remains inconclusive as to which virus predominantly contributed.^[16]^ In contrast, our analysis has delineated a negative causal association between levels of Herpes simplex virus 1 mgG-1 antibody levels and miscarriage (IVW: OR 0.923; 95% CI 0.864–0.987; *P* = .019). The presence of HSV-1 mgG-1 antibodies indicates that an individual has been infected with the virus and that their immune system has responded. Over time, the immune system may mature and adapt to control the virus more effectively, reducing the inflammatory response that can lead to miscarriage. Based on a comprehensive analysis, it is concluded that HSV-1, a highly prevalent virus globally, can contribute to an increased risk of miscarriage. However, the presence of antibodies against the virus in the body provides a certain degree of protection for the pregnant population.

In another Korean study, it was found that pregnant women who tested positive for HSV-2 antibodies had a higher rate of miscarriage, which contrasts with our research findings.^[17]^ We did not identify a causal relationship between HSV-2 infection and miscarriage, possibly due to the fact that in their study, most women also tested positive for rubella, varicella-zoster, and hepatitis B viruses, affecting the reliability of the conclusions.^[17]^

Moreover, preliminary observational studies with limited sample sizes have indicated the presence of elevated levels of IgG antibodies against human cytomegalovirus in patients who have experienced miscarriages.^[42]^ In in vitro experiments, it has been demonstrated that cytomegalovirus is able to replicate within trophoblasts, eliciting inflammatory responses and subsequently leading to heightened cellular apoptosis.^[43]^ Nevertheless, our comprehensive magnetic resonance analysis did not reveal a causal relationship between human cytomegalovirus infection and its associated antibodies with miscarriage.

Additionally, this study has revealed 2 EB virus-related antibody-mediated immune responses that may serve as protective factors against miscarriage. We hypothesize that following an EB virus infection, the immune system begins to respond. Over time, the immune system may mature and become more efficient at controlling the virus, thereby reducing the risk of inflammatory responses that could lead to miscarriage. Furthermore, the potential causal link between human herpesvirus 7 type U14 antibody levels and miscarriage further suggests the role that herpesvirus 7 may play in the mechanisms underlying miscarriage. However, research in this area is limited, with occasional mentions suggesting that these viruses may be associated with adverse pregnancy outcomes.^[41,44]^

Lastly, to our knowledge, there have only been 2 previous studies on the relationship between human herpes virus 6 and fetal miscarriage, with conflicting results.^[45,46]^ In our study, we found a causal relationship between anti-human herpes virus 6 E1A IgG seropositivity and miscarriage, which reached significance in 2 datasets, indicating a high level of credibility. This finding warrants further exploration and investigation.

#### 4.1.2. Polyomavirus

In this study, we mainly investigated the causal relationship between antibodies related to JCPyV, BKPyV, MCPyV infection, and miscarriage.

Initially, JCPyV is prevalent in the population, with approximately 50% of healthy individuals carrying IgG antibodies against it.^[47]^ Research has demonstrated the presence of JCPyV DNA in human seminal fluid and urine samples from pregnant women, suggesting the potential for transmission through sexual contact to the uterus/embryo.^[48]^ Understanding the transmission pathway is crucial for evaluating the risk of JCPyV infection during pregnancy. Consistent outcomes were derived from the MR analysis of our 2 miscarriage GWAS datasets, revealing a causal link between anti-polyomavirus 2 IgG seropositivity and miscarriage, underscoring the robustness of the findings. Nevertheless, this conclusion contrasts with the findings of Tagliapietra et al, who did not identify a correlation between the variables, indicating the necessity for further investigations.^[48]^

Furthermore, there is limited understanding of the potential role of BKPyV in adverse pregnancy outcomes. The few data available are discordant. Some studies have found BKPyV detected in samples from miscarriages due to chorioamnionitis, suggesting a potential role of this human polyomavirus in miscarriages.^[22]^ However, in our study, we found no causal relationship between BKPyV infection and miscarriage. This conclusion is consistent with the findings of Tagliapietra et al, where the detection rate and viral DNA load of BKPyV showed no significant differences between spontaneous miscarriage and voluntary termination of pregnancy groups.^[48]^

MCPyV is a small DNA tumor virus that is commonly present in humans. MCPyV establishes clinically asymptomatic lifelong infection in individuals with healthy immune function. This study found a causal relationship between anti-MCPyV IgG positivity and miscarriage, with consistent conclusions drawn from GWAS data analysis in 2 miscarriage cohorts. Additionally, significant results were also obtained in subsequent meta-analyses. However, Mazziotta et al also identified a role of MCPyV in miscarriage, but contrary to this, they found that the optical density of MCPyV IgG antibodies in serum samples of women with spontaneous miscarriage was lower compared to those with elective termination of pregnancy.^[49]^ This may require further research with larger sample sizes for support.

### 4.2. Protozoa and bacteria

#### 4.2.1. Toxoplasmosis

Toxoplasmosis infection is highly prevalent worldwide. In a study conducted in London, 17.32% of 2610 samples tested positive for Toxoplasma antibodies.^[50]^ While most patients are asymptomatic, individuals with compromised immune systems are at risk of severe illness. Pregnant women infected during pregnancy can vertically transmit the infection.^[51]^ In our study, we did not find a significant correlation between Toxoplasma infection and miscarriage. Although observational studies have suggested a higher rate of Toxoplasma IgG seropositivity in women who have experienced miscarriages, the lack of a control group limits a comprehensive understanding of the relationship between Toxoplasma infection and miscarriage.^[19,20]^

#### 4.2.2. Chlamydia trachomatis

*C trachomatis*, the most common sexually transmitted infections globally, is a known risk factor for ectopic pregnancy and preterm birth.^[52,53]^ However, the relationship between *C trachomatis* infection and miscarriage remains highly debated.^[11,12,15]^ This study found a causal relationship between anti-chlamydia trachomatis IgG seropositivity and miscarriage (IVW: OR 0.955; 95% CI 0.918–0.994; *P* = .023), but this result has not been validated in other datasets. The immune response triggered by *C trachomatis* infection, serving as a protective factor against miscarriage, reveals the role that *C trachomatis* plays in the process of miscarriage. Persistent asymptomatic *C trachomatis* infection spreading to fetal tissues or the endometrium can trigger miscarriage.^[11]^ Some patients exhibit *C trachomatis* seropositivity without detectable *C trachomatis* DNA, suggesting that miscarriage may occasionally be induced by past chlamydial infections or by persistent *C trachomatis* antibodies that may interfere with embryonic antigens.^[9]^

### 4.3. Strengths and limitations

Pregnancy represents a sophisticated biological process, orchestrated by a symphony of cellular interactions and regulated by a network of complex mechanisms. In an endeavor to elucidate the contributory factors leading to miscarriage, this investigation examines the interplay between infections, the consequent antibody-mediated immune responses, and the incidence of miscarriage.

Our study has notable strengths. Firstly, we believe we’re the first to deeply explore the potential causal relationships between various pathogen infections and miscarriage using a two-sample MR analysis. This work could significantly influence clinical decisions and public health strategies. Secondly, our study combines GWAS data from 3 miscarriage cohorts for a comprehensive analysis. The findings have been rigorously validated through tests for heterogeneity, horizontal pleiotropy, and leave-one-out sensitivity analysis, and further confirmed via meta-analysis, ensuring high stability and reliability of our conclusions.

Our study also has some limitations. While the study principally employs MR analysis, it stops short of probing the intricate molecular mechanisms at play. Additionally, the dataset underpinning the study captures the aftermath of pathogen infections through the lens of antibody-mediated immune responses, yet it does not extend to the evaluation of mRNA and protein expressions of the pathogens within tissue samples. As such, while the study offers insights into the repercussions of pathogen infections, it cannot conclusively dismiss the potential for persistent pathogen antibodies to disrupt embryonic antigens. Despite the reliance on somewhat antiquated references concerning pathogen infections and miscarriage, these seminal works have furnished us with a trove of fundamental data. Such information paves the way for a more profound comprehension of the possible associations between pathogenic infections and miscarriage, without undermining the substantial import of our analytical conclusions. Moreover, considering that the core data for this study emanate from European cohorts, the extrapolation of the findings to diverse ethnicities or populations should be approached with caution. Variabilities in genetic makeup, lifestyle choices, and environmental exposures could lead to disparities in outcomes across different demographic groups. Lastly, in response to existing controversies, we employed the advanced methodology of MR in an attempt to address these issues and to present our findings comprehensively. Although this approach has been proven to play a significant role in exploring the associations between exposures and outcomes, we did not validate our findings through in vivo or in vitro experiments (as validation was not our primary objective). Therefore, our results are not flawless, and further validation with large-scale controlled experiments will be necessary in the future.

## 5. Conclusion

Employing rigorous MR analysis, this study has uncovered a causal link between infections caused by certain pathogens, namely, members of the herpes virus family, *C trachomatis*, and the MCPyV, and the occurrence of miscarriage. In light of these findings, it is recommended that healthcare professionals incorporate the screening for these specific antibodies into their clinical assessments to better manage the risk of miscarriage.

## Author contributions

**Conceptualization:** Jie Zhou, Jiekai Yin.

**Data curation:** Jie Zhou.

**Formal analysis:** Jie Zhou.

**Funding acquisition:** Yixin Xu.

**Investigation:** Jie Zhou.

**Methodology:** Jie Zhou.

**Resources:** Haitao Wang.

**Software:** Yixin Xu, Haitao Wang.

**Supervision:** Jiekai Yin, Yixin Xu.

**Validation:** Jiekai Yin, Yixin Xu.

**Visualization:** Haitao Wang.

**Writing – original draft:** Jie Zhou.

**Writing – review & editing:** Jiekai Yin.

## Supplementary Material


