# Supplementary figures and images for: Unveiling the truth: Pathogen infections linked to miscarriage: A STROBE-Compliant Mendelian randomization study
Source: Medicine (Baltimore). 2024 Nov 22;103(47):e40627. doi: 10.1097/MD.0000000000040627 (PMC11596664; doi:10.1097/MD.0000000000040627)

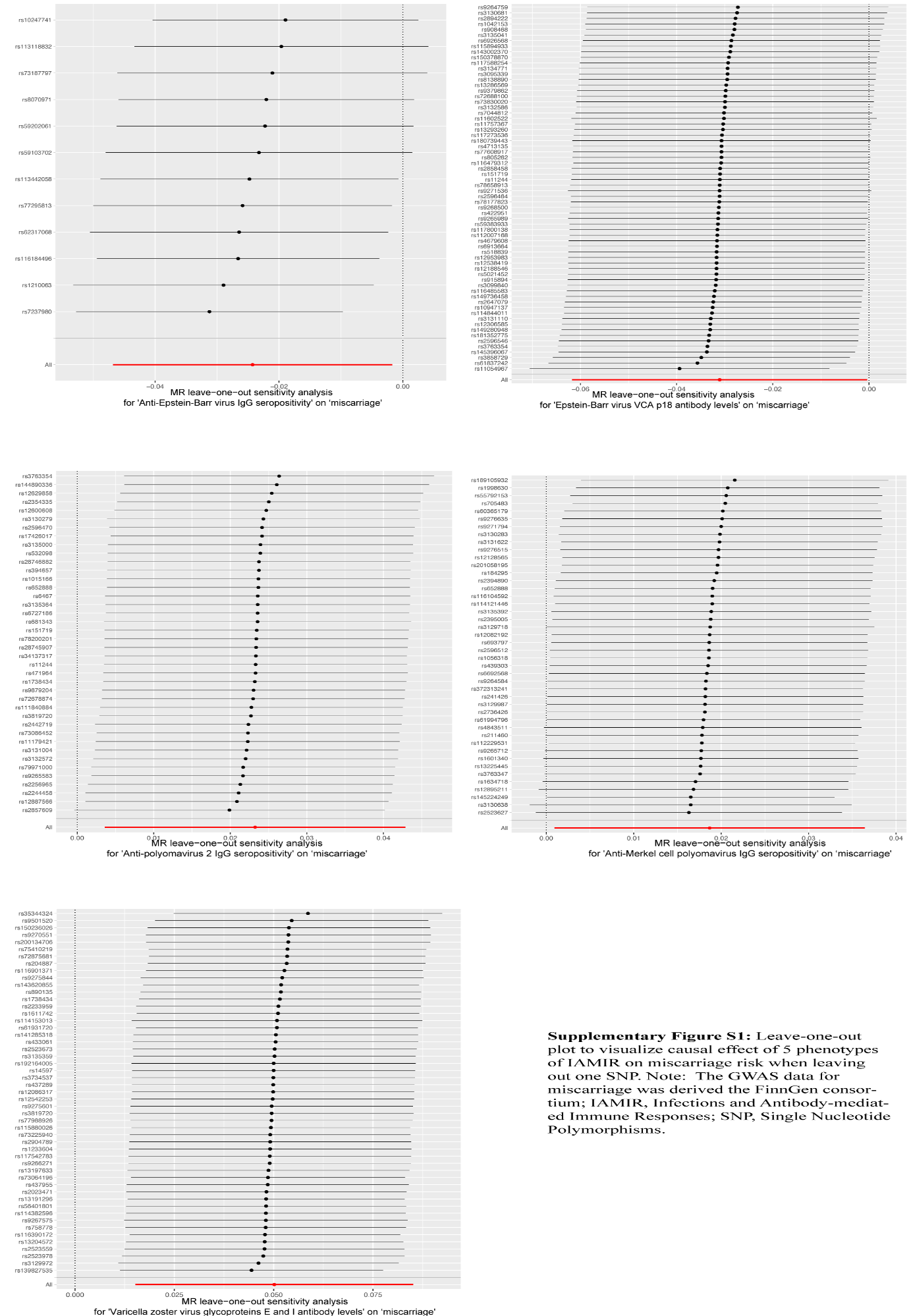


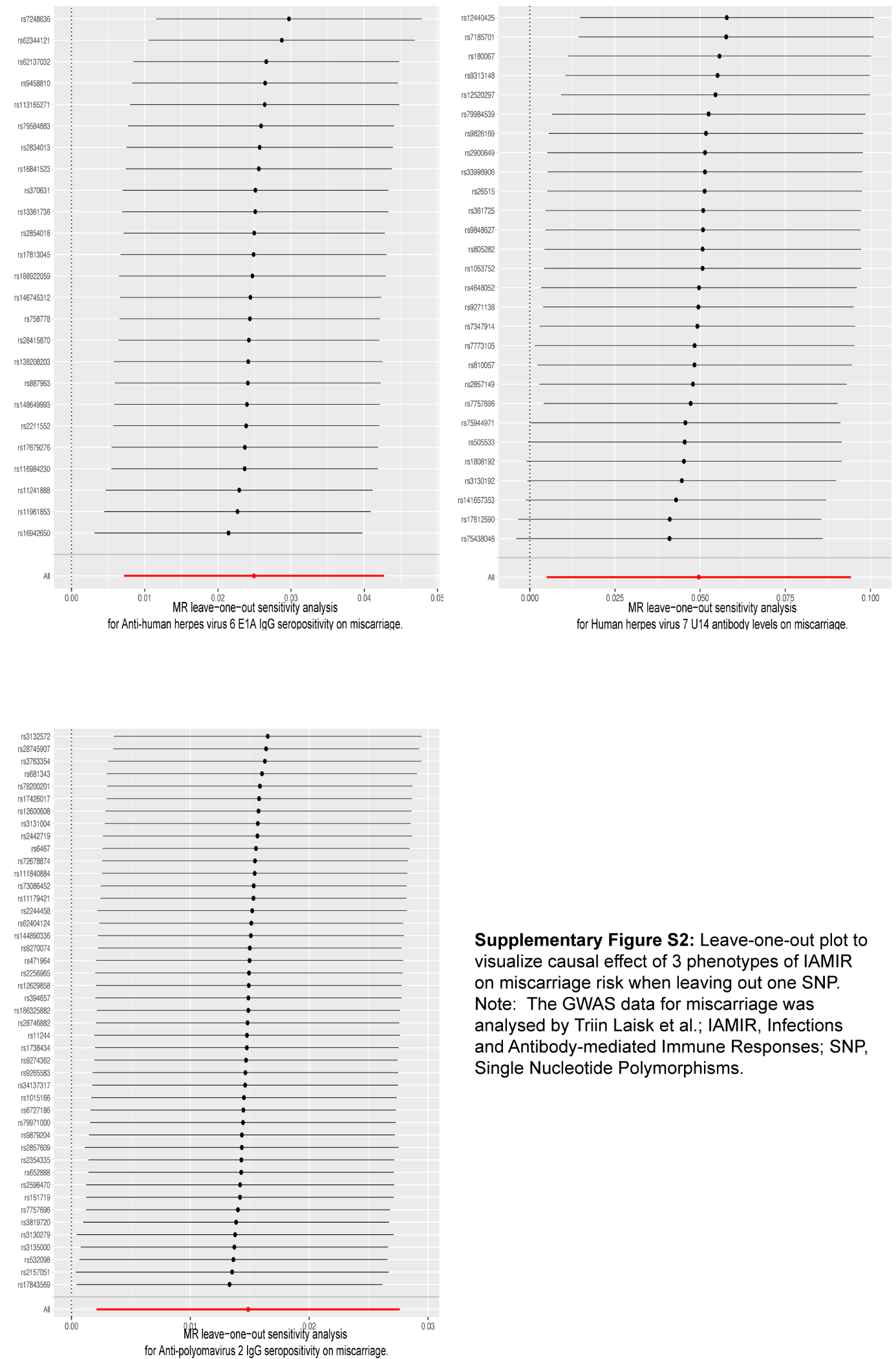


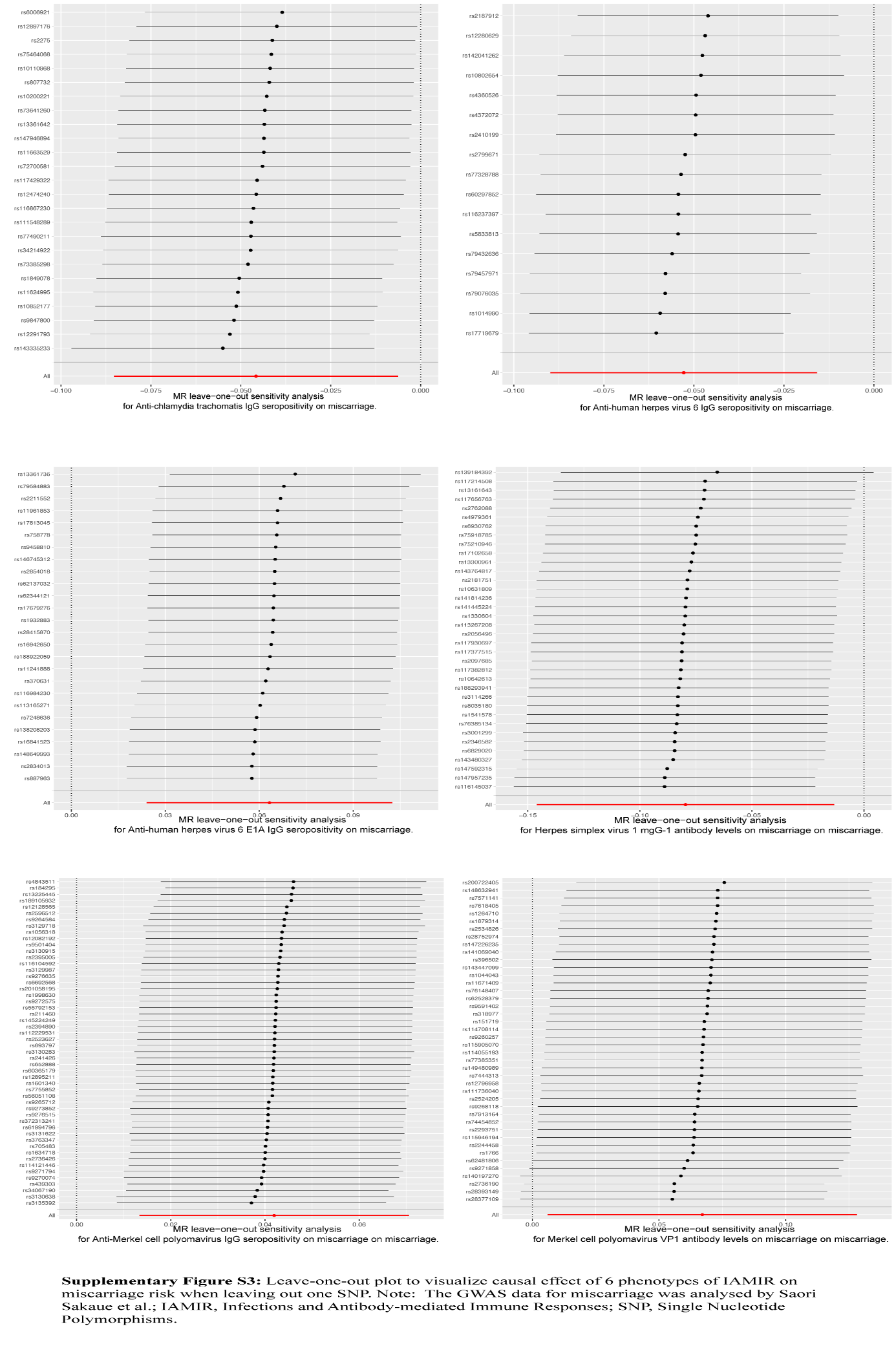

Supplement: Supplementary file 2 [file medi-103-e40627-s002.docx]
